# Learning-based real-time imaging through dynamic scattering media

**DOI:** 10.1038/s41377-024-01569-0

**Published:** 2024-08-16

**Authors:** Haishan Liu, Fei Wang, Ying Jin, Xianzheng Ma, Siteng Li, Yaoming Bian, Guohai Situ

**Affiliations:** 1 Shanghai Institute of Optics and Fine Mechanics, Chinese Academy of Sciences, Shanghai, 201800 China; 2 Center of Materials Science and Optoelectronics Engineering, University of Chinese Academy of Sciences, 100049 Beijing, China; 3 Department of Engineering Science, University of Oxford, Oxford, UK; 4 Hangzhou Institute for Advanced Study, University of Chinese Academy of Sciences, Hangzhou, 310024 China

**Keywords:** Imaging and sensing, Atmospheric optics

## Abstract

Imaging through dynamic scattering media is one of the most challenging yet fascinating problems in optics, with applications spanning from biological detection to remote sensing. In this study, we propose a comprehensive learning-based technique that facilitates real-time, non-invasive, incoherent imaging of real-world objects through dense and dynamic scattering media. We conduct extensive experiments, demonstrating the capability of our technique to see through turbid water and natural fog. The experimental results indicate that the proposed technique surpasses existing approaches in numerous aspects and holds significant potential for imaging applications across a broad spectrum of disciplines.

## Introduction

Classical image formation theory relies on a fundamental assumption: the spatial-spectral information carried by light is not excessively distorted as it propagates all the way from the object to the imaging system^1^. Otherwise, the captured image can be severely degraded owing to the presence of scattering noise, which manifests as speckle grains under coherent light illumination or leads in a reduction in image contrast under incoherent light illumination^2^. Imaging through scattering media, such as biological tissues, haze, fog, and turbid water, is scientifically and technically important, yet it poses a considerable challenge. That challenge has long been recognized as one of the longstanding problems in the field of optical imaging.

Traditional methods for addressing this issue typically aim to either isolate the early-arriving light components from the later-arriving, multiply-scattered ones^3–10^ or to improve the signal-to-noise ratio. In terms of selection techniques, groundbreaking research has led to the creation of gating methods that capitalize on the Kerr effect in nonlinear media^4–8^, as well as the coherence^9^ and polarization^10^ characteristics inherent to the light. For enhancing the early-arriving light, one strategy is to employ an illumination source that is well-suited to the scattering properties of the medium, particularly if these properties are spectrum-dependent^11^. Conversely, to mitigate the noise from multiple scattering, one could exploit the absorptive properties of the medium^12^ or implement spatial filtering within a 4f system^13^. Nonetheless, since the ballistic light decays exponentially with the optical thickness of the scattering medium^4,13^, techniques that depend on it are inherently limited in their capacity for image quality restoration and depth penetration.

Computational techniques can leverage the performance of imaging through scattering media by utilizing not only the early-arriving light but also part of the late-arriving scattered light^14–33^. Among these techniques, deep learning(DL) has garnered increasing interest^24–26,29–33^ due to its capacity of addressing challenging inverse problems^34^. As a class of data-driven algorithms, the performance of DL is heavily dependent on the quality of the training dataset^35^. Although there are physics-enhanced neural networks (PhysenNet) that do not require training data and instead rely on the physical principle of the forward model^36–40^, general forward physical models are often too complex to formulate^41^ for imaging through scattering media, with exception of the simpler cases involving thin scattering layers^30^.

However, existing studies in this area are more conceptual than practical. There are at least two reasons for this. The first pertains to the nature of the scattering media: Existing studies have been conducted in artificial scattering media such as ground glasses^24,25,30,31,33^, polystyrene slabs^26^, or fat emulsion suspension^29,32^. These media are either homogeneous on a macroscopic scale or at least quasi-static during the acquisition process. In contrast, practical applications involving scattering media like fog, turbid water, and in situ biological tissues exhibit significant different properties: they are non-static and inhomogeneous^2^. This discrepancy results in a challenging situation where each pair of data in the training set - the acquired scattered patterns and the corresponding ground truth images – may be associated with a distinct transmission matrix^42^ under coherent light illumination. A deep neural network (DNN) trained on such an inconsistent dataset may fail to converge to a model that accurately maps the labels.

This issue also feeds into the second concern, which is the creation and acquisition of the training dataset. In existing studies, ground truth images have been sourced from public datasets like MNIST^26^, CIFAR, and ImageNet^43^. For demonstrations, these images were typically displayed on a spatial light modulator (SLM) or a digital micromirror device (DMD). In order to optically acquire the ground truth images, these devices were illuminated by collimated, coherent light from the object side, bypassing the scattering medium^24–26,29,31–33^. This implies an invasive operation, which is impractical since, in such cases, one could directly image the objects hidden inside or behind the scattering medium. Furthermore, using SLM/DMD for this application presents another issue: their reflectance characteristics differ significantly from those of real-world objects. As a result, the ground truth images captured in this manner may not accurately reflect the optical properties of the real-world scenes.

Here, we propose a comprehensive learning-based method named DescatterNet for incoherent imaging through non-static and inhomogeneous scattering media. The most significant advantage that set DescatterNet apart from other existing learning-based methods, such as HNN^26^, MulScaleCNN^29^, Unet^44^, AttentionUNet^45^, and SwinIR^46^, is its ability to handle natural and complex scenes effectively. We demonstrate the effectiveness of the proposed DescatterNet through extensive experiments conducted both indoors and outdoors. The results show that DescatterNet achieves outstanding performance when compared to traditional image enhancement methods^47–51^, particular in terms of reconstructed image quality, inference speed, and memory consumption.

## Results

**Fig. 1:**
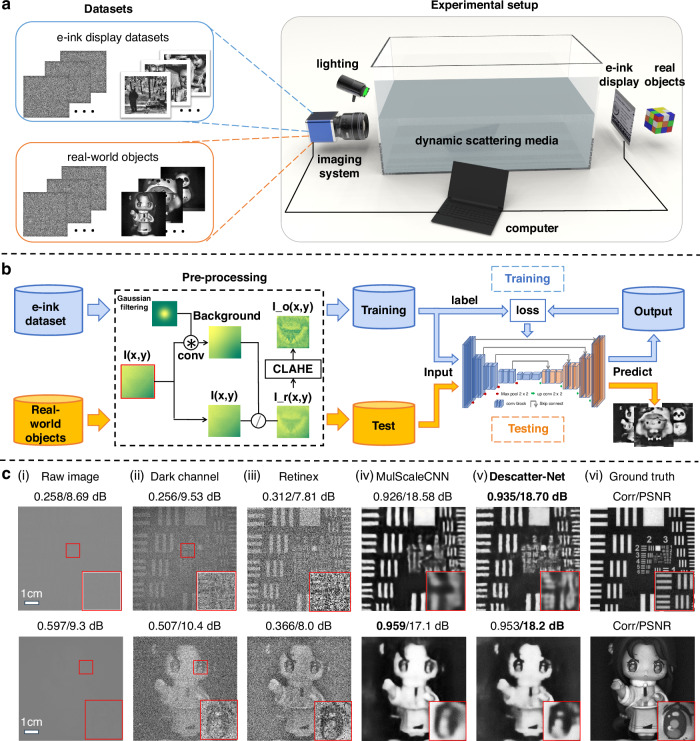
Overview of the DescatterNet. **a** Indoor experimental setup for capturing the dataset to train and test the proposed method. **b** Flowchart of the training and testing processes. **c** Representative experimental results. (i) The raw image, and the images reconstructed by (ii) the dark channel prior algorithm, (iii) the Retinex-based algorithm, (iv) MulScaleCNN, and (v) the proposed DescatterNet. vi The ground truth images for comparison

The overview of our DescatterNet is illustrated in Fig. 1. We address three critical challenges: acquiring “real” scattering datasets, ensuring generalization to previously unseen real-world objects (including outdoor scenes), and identifying the optimal neural network architecture. Initially, we collected thousands of “real” scattered-clear image pairs under various scattering conditions using our custom experimental setup [Fig. 1a]. Subsequently, to enhance the generalization of our model to real-world objects and outdoor scenes that are not encountered during training, we propose a preprocessing method to bridge the domain gap between across different scattering conditions [Fig. 1b]. Lastly, we conducted a thorough exploration of the superior neural network by optimizing the network architecture and comparing it with several alternatives detailed in Fig. 1c and Table 1. For in-depth explanation, please refer to the Methods and Materials section.

**Table 1:** Performance comparison of 6 neural network architectures in terms of *N*_*para*_ (model size), FLOPs (computational complexity), FPS (inference speed) and Corr and PSNR (image quality)

Index	Method	*N*_*para*_	FLOPs	FPS(RTX3090)	Corr	PSNR
1	**DescatterNet**	1.94 M	10.59 G	**338.62**	**0.8488**	**18.00**
2	HNN^26^	1433.12 M	16.86 G	38.60	0.6946	15.25
3	MulScaleCNN^29^	1.41 M	6.38 G	80.43	**0.8492**	17.93
4	Unet^44^	31.04 M	167.51 G	65.02	0.8432	17.80
5	AttentionUNet^45^	34.88 M	203.83 G	49.95	0.8468	17.83
6	SwinIR^46^	0.14 M	33.97 G	1.15	0.8218	17.11

The bold font emphasizes the superior performance of the method over the other five in the comparative study

### Recovery of previously unseen real-world objects

s shown in Fig. 1b, we trained our DescatterNet on the dataset in which the ground truth images were displayed on an e-ink display. We demonstrate that the trained DescatterNet can be directly applied to recover previously unseen real-world objects from the corresponding raw images (i.e., the scattered patterns) captured through the same scattering medium.

In this experiment, the scattering medium was the tank of fat emulsion with an optical thickness of 5.51 as measured^26^. In such a highly scattering environment, the raw images exhibit extremely low contrast, obscuring all visual information within the scattered light [Fig. 1c(i)]. Traditional image contrast enhancement algorithms, such as dark channel prior (DCP)^47^and Retinex^49^, were used to restore the images, with the results displayed in Fig. 1c(ii) and Fig. 1c(iii), respectively. One can see that the images reconstructed by conventional algorithms like DCP and Retinex are noisy, with associated peak signal-to-noise ratio (PSNR) values of less than 10 dB. In contrast, the proposed DescatterNet significantly outperforms the other methods [Fig. 1c(v)].

For a visual impression, we zoom in on the central region of the raw image of the USAF resolution chart and the same regions of the images reconstructed by the four methods. Obviously, the dark channel prior algorithm performs the poorest because it depends on the shadow cast on the raw images^47^. Noise also evident in the image reconstructed by the Retinex algorithm, as it relies on the estimation and subsequent subtraction of the illumination pattern^51^, which is randomly distributed in our scenario. The images reconstructed by MulScaleCNN^29^ and the proposed DescatterNet are of much higher quality, both in terms of PSNR and Correlation Coefficient (Corr) with respect to the ground truth, as shown in Fig. 1c(vi). It is clear that the images reconstructed by DescatterNet retain more structural details, especially in high-resolution regions (highlighted with a red box) compared to the those reconstructed by MulScaleCNN^29^. The loss of high-resolution information in the image reconstructed by MulScaleCNN is paimarily attributed to its network structure that the downsampling deconvolution layers are placed upstream of the feature extraction branches.

### Upper limit of descattering performance

Now we proceed to assess the performance of the proposed DescatterNet with respect to the optical thickness of the scattering medium, which, in our indoor laboratory experiment, can be controlled by volume *V* of fat emulsion dropped in the tank of water.

For this analysis, we collected a set of ground truth images (presented on the e-ink display) and their corresponding scattered patterns (raw images). We randomly selected five distinct scattering strengths for our study, corresponding to different volumes of fat emulsion (*V* = 1.8 ml, 2.4 ml, 2.8 ml, 3.2 ml, and 3.6 ml, respectively). Then we trained five distinct DNN models with identical architectures on these five datasets, and used them to reconstruct images from the captured raw images.

**Fig. 2:**
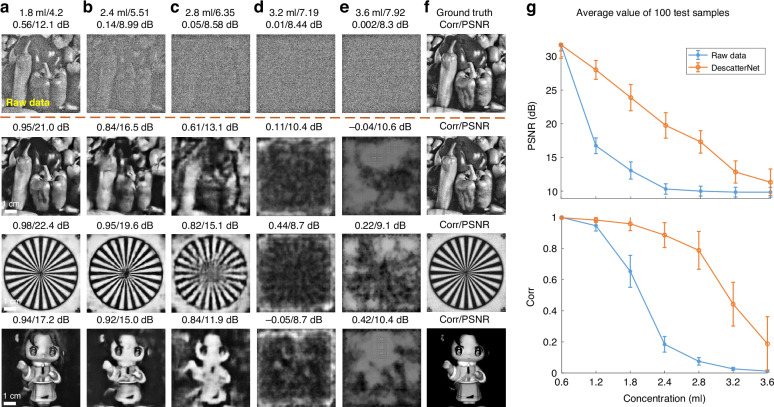
Descattering performance under varying concentration condition. The images in the first row are the raw scattered patterns captured by the camera through a tank of fat emulsion with V equal to **a** 1.8 ml, **b** 2.4 ml, **c** 2.8 ml, **d** 3.2 ml, and **e** 3.6 ml, respectively, and **f** is the ground truth. The images in the second row are the image reconstructed using the proposed DescatterNet. It is observable that the quality of the recovery images deteriorates with increasing concentration, and the images become completely corrupted when the volume *V* exceeds 2.8 ml. The images in the third and fourth rows provide additional two sets of examples. **g** The PSNR and Corr values associated with the raw and reconstructed images at different concentrations

As shown in Fig. 2, the representative images demonstrate that the objects can be clearly reconstructed when the volume *V* is low. The reconstructed images gradually become distorted and noisy as the scattering strength of the medium increases. The images eventually become entirely corrupted when *V* falls between 2.8 ml and 3.2 ml, which we surmise to be the upper limit for the effectiveness of our proposed method.

Regrettably, we have not yet determined the precise critical value of *V*. There are two main reasons for this. Firstly, the precise knowledge of the critical value does not significantly influence the understanding or application of the proposed DescatterNet. In essence, this is not the physical limitation but rather an engineering constraint of the proposed method. We anticipate that the limit can be improved by employing a superior camera with higher dynamic range, lower noise level, larger quantum efficiency, optimizing the network structure, or utilizing a powerful illumination light source. Secondly, the proposed DescatterNet fundamentally relies on the utilization of the early-arriving light, which are typically overwhelming by much stronger multiple scattered light. Should one be able to devise an acquisition technique that can separate these light components and develop a smarter method to harness the information they contain, we believe that the limit could be improved. This suggests that the current constraints are not insurmountable and that advancements in technology and methodology could extend the capabilities of DescatterNet.

### Generalization

We proceed to assess the generalization capabilities of the proposed DescatterNet. This analysis will be conducted along two distinct dimensions:

- Cross-concentration generalization: This involves evaluating how well the DescatterNet performs when applied to the same type of scattering medium, but with varying concentrations or densities. The goal is to determine if the DescatterNet can effectively adapt to and reconstruct images through media with different level of scattering strengths without requiring retraining for each specific concentration.
- Cross-media generalization: This aspect of analysis will test the DescatterNet’s ability to generalize across different types of scattering media. The objective is to understand if the DescatterNet can maintain high performance when faced with diverse scattering characteristics that may be fundamentally different from those encountered during the training phase.

By examining these two factors of generalization, we aim to establish the robustness and flexibility of DescatterNet in handling a wide range of real-world scenarios involving various scattering conditions.

### Cross-concentration generalization

**Fig. 3:**
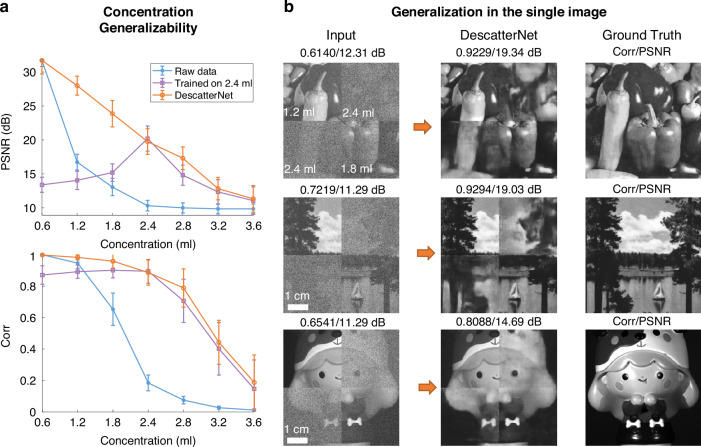
Cross-concentration generalizability assessment. **a** The blue curve depicts the PSNR and Corr values of the raw scattering images across various concentrations. The purple curve indicates the performance of the DescatterNet model trained solely on the 2.4 ml concentration dataset. In contrast, the orange curve shows the performance of the DescatterNet model trained on a mixed dataset, highlighting its adaptability to different concentrations. **b** The reconstructed images for a single image subjected to different scattering strengths. The composite images, which integrate scattering patterns of multiple concentrations, are processed by the DescatterNet. This demonstration verifies the model’s proficiency in image restoration across a spectrum of scattering conditions

Here we provide a single illustrative example to showcase the cross-concentration generalization. The blue curves in Fig. 3a represent the similarity of the captured scattered pattern (raw data) and the ground truth images, calculated as an average over 100 different test samples. Both the correlation coefficient (Corr) and PSNR metrics indicate that the scattered patterns closely resemble the corresponding ground truth images when the concentration of the emulsion suspension is low. The reconstructed image progressively becomes noisier as the concentration increases, and completely submerged in noise when the concentration reaches around 2.4 ml [Fig. 2a].

In contrast, the purple curve shows the performance of a DescatterNet trained solely on a dataset with the concentration of *V* = 2.4 ml. The model demonstrates an improvement in image reconstruction from raw data across various concentrations, although images at low concentration exhibit some degradation due to residual noise and loss of fine structures. This issue can be dramatically mitigated by training the DescatterNet on a mixed dataset that contains both low and high concentration data, as suggested by the comparatively flat orange curves in Fig. 3a.

The cross-concentration generalization capability of the proposed DescatterNet can be further exemplified by its ability to reconstruct images from “virtual” scattered patterns synthesized from experimentally acquired raw data at different concentrations. Three examples of such virtual scattered patterns are shown in Fig. 3b (labeled as Input): They were synthesized from four regions captured through the tank of fat emulsion with different concentrations (top-left: 1.2 ml, top-right: 2.4 ml, bottom-left: 2.4 ml, bottom-right: 1.8 ml). It is evident that all four regions are well recovered in a single inference, with a marked improvement in mean Corr/PSNR from 0.66/11.63 dB to 0.89/17.69 dB.

### Cross-media generalization

Now we proceed to examine how well a DescatterNet model, trained on a dataset from a fat emulsion, can reconstruct images from scattered patterns captured through a milk suspension and artificial fog.

The milk suspension was prepared by applying drops of fresh milk to a tank of pure water, as shown in Fig. 1a. The artificial fog was generated by using an ultrasonic nebulizer (model SG-06D/10D), which filled the entire tank with fog.

**Fig. 4:**
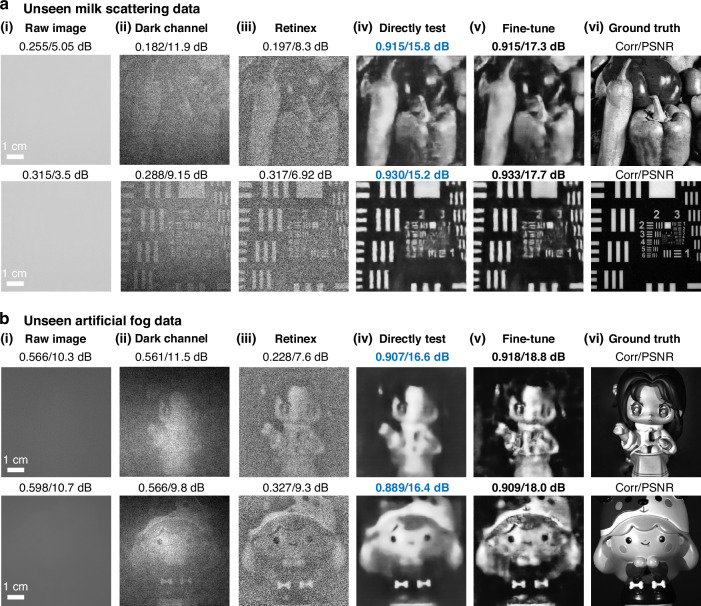
Cross-media generalization assessment. Reconstructed images for **a** milk and **b** artificial fog scattering data. In **a** and **b**, (i), The raw scattering images captured by the camera, and the images reconstructed using (ii) dark channel prior method, (iii) our preprocessing Retinex-based method, (iv) DescatterNet trained on fat emulsion dataset, (v) DescatterNet with fine-tune using milk/artificial fog scattering data. (vi) The ground truth images for comparison

It is known that different scattering media consist of micro particles of varying types and sizes^2^, leading to unique scattering characteristics. This affects how object information propagates through the scattering medium and the resulting formation and statistics of the scattering patterns. This variation is clearly seen by comparing the experimentally acquired raw data in Fig. 1c (fat emulsion), Fig. 4a (milk), and Fig. 4b (artificial fog), all captured under identical illumination condition and camera setting.

The high dynamic range of the sCMOS camera (PCO Edge 4.2) allows the raw data to capture a small amount of ballistic and snake light that carries information about the objects. Employing a Retinex-based method (details can be found in the Method and Materials section), one can reconstruct images from the raw data, as shown in Fig. 4a(iii), b(iii). However, these images are quite noisy, with a correlation coefficient of around 0.2 and a PSNR of around 8 dB.

When we apply the proposed DescatterNet to the reconstructed noisy images, we find that despite being trained on a fat emulsion dataset, the DescatterNet can significantly enhance the degraded images obtained through other scattering media [Fig. 4a(iv), b(iv)], with the Corr/PSNR increased dramatically from around 0.2/8 dB to 0.9/16 dB.

Furthermore, the performance of the DescatterNet can be optimized even more by fine-tune the trained model with an additional dataset specific to the scattering media in question, such as milk and artificial fog. This fine-tuning process, as illustrated in Fig. 4a(v), b(v), leads to a further enhancement in the quality of the reconstructed images, showcasing the method’s adaptability and potential for practical use across different scattering media.

### Outdoor experiment results

To assess the practical performance of our method, we demonstrate that the proposed DescatterNet, trained on a fat emulsion dataset, can be directly used for outdoor imaging through natural fog. The optical system used for the outdoor experiments is depicted in Methods and Materials section.

**Fig. 5:**
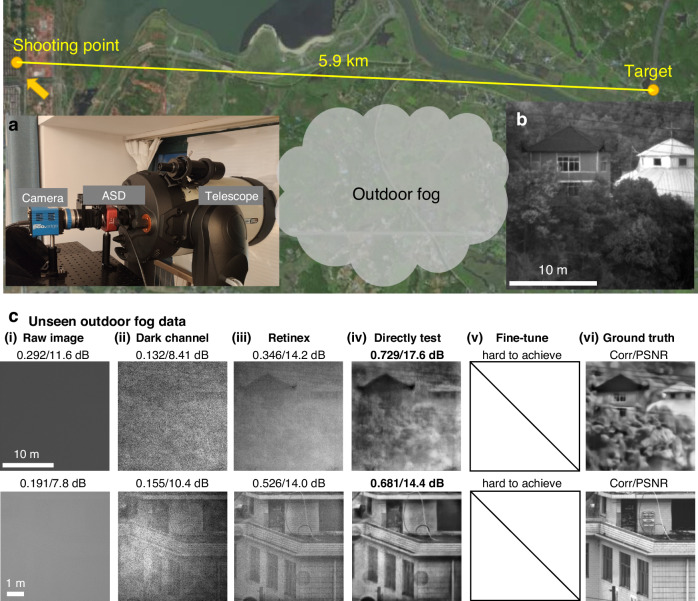
Outdoor experimental results. **a** Outdoor imaging system. **b** The targets to be imaged in our outdoor experiment. **c** The reconstructed images from various methods. In **c**, (i) The raw images captured under scattering conditions and the reconstructed images using (ii) dark channel prior method, (iii) Retinex-based algorithm, (iv) DescatterNet trained on indoor dataset, and (v) fine-tune DescatterNet (which is hard to achieve in this case) and (vi) The ground truth images, serving as references for comparison

Figure 5 presents the main results from the outdoor experiments. For analytical purpose, we captured images of the same scenes – houses in forest, and a villa – under pristine weather conditions, which we used as the ground truth [Fig. 5c(vi)]. On various foggy days, we recorded the raw scattered patterns [Fig. 5c(i)] and reconstruct the images using the DescatterNet. The reconstructed images shown in Fig. 5c(iv) exhibit coefficient correlation and PSNR values of approximately 0.7 and 17 dB for the houses in the forest, and 14 dB for the villa, respectively. It is noteworthy that neither the scenes nor the scattering media were seen in the training dataset; nonetheless, the results suggest that the proposed DescatterNet is capable of capturing relevant information. Given the challenge of collecting thousands of scattered-clear image pairs under outdoor conditions, there are no fine-tuning results, as shown in Fig. 5c(v), which illustrates one of the longstanding obstacles hindering the practical application of deep learning technology. Naturally, the quality of the reconstructed images in this scenario may not match those achieved in a controlled laboratory environment. However, the DescatterNet still delivers superior performance compared to the conventional non-learning-based method we proposed in a previous study^52^.

## Discussion

In this section, we discuss the accuracy of our hypothesis and their contribution to the optimal reconstruction performance of our method through various dynamic scattering media in real time.

### The functionality of the e-ink display

Our hypothesis posits that the e-ink display can effectively simulate real-world objects, which is crucial for creating a representative training dataset. Unlike SLM or DMD, which have reflectivity that differs from natural scenes, the e-ink display’s Lambertian surface provides a more realistic simulation with consistent brightness from all viewing angles^53^.

To validate this hypothesis, we conducted a comparative study between the e-ink display and an SLM. By swapping the e-ink with an SLM (Pluto 6001, HoloEye Photonics AG) in our setup, we maintained all other conditions constant^29^. Despite the SLM’s small field-of-view, we resized images to match this, resulting in two datasets for training the DescatterNet. The performance is evaluated by the correlation coefficient and the PSNR.

**Fig. 6:**
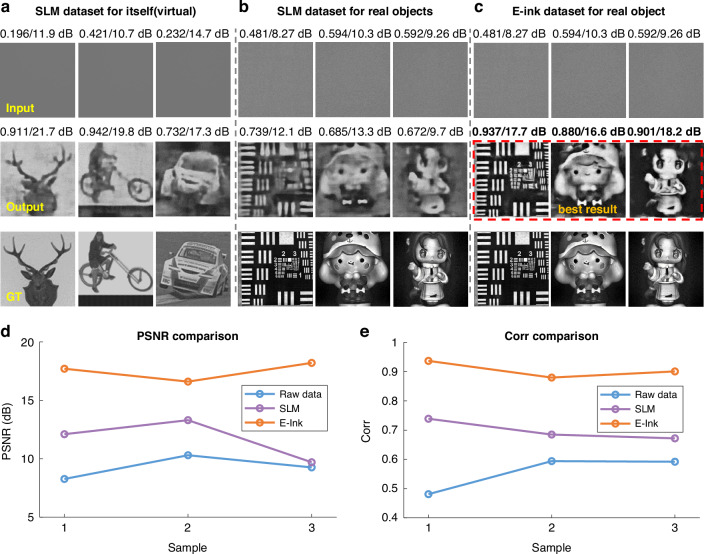
Performance comparison between SLM and E-ink display. **a** The raw images of virtual objects displayed on the SLM and the corresponding images reconstructed by a DescatterNet trained on an SLM dataset. **b** The raw images of real-world objects and the corresponding images reconstructed by a DescatterNet trained on an SLM dataset. **c** The raw images of real-world objects and the corresponding images reconstructed by a DescatterNet trained on an e-ink dataset. **d** the PSNR and **e** the Corr values associated with the raw image of the three real-world objects, and the images reconstructed by a DescatterNet trained on SLM dataset and e-ink dataset, respectively

The results shown in Fig. 6 provides a clear illustration of the comparative performance between the DescatterNet models trained on these two datasets. The first row of images presents the raw scattering patterns as captured by the camera, with the second row showcasing the reconstructed images, and the third row displays the ground truth images for reference. It is evident that a DescatterNet trained on SLM dataset can effectively reconstruct images that were displayed on the same SLM [Fig. 6a]. The correlation coefficient for simple objects are notably high, exceeding 0.9, and even complex object like a race car achieve a Corr greater than 0.7, consistent with the findings from our previous study^29^. However, the same model, when tasked with reconstructing images of real-world objects after replacing the SLM with such objects, such as the USAF target and China dolls, exhibits significant decline in performance, with Corr falling below 0.7, as shown in Fig. 6b.

The contrast is strikely evident when compared to the performance of a DescatterNet trained on an e-ink dataset. As shown in Fig. 6c, it demonstrates a remarkable ability to reconstruct images of real-world objects with high fidelity, as indicated by Corr values approximately equal to 0.9. This suggest that the e-ink display, with its more natural reflectivity characteristics, provides more suitable data for training. Readers can refer to the Supplementary Video for a more comprehensive visual representation of the results.

### The effect of the preprocessing method

A pivotal factor contributing to the robust generalization and excellent results of our method is the implementation of an effective preprocessing strategy. Captured images are subject to a myriad of factors, including variations in concentrations, types of scattering media, lighting conditions, and optical systems, which can lead to diverse scattered patterns even for a constant scene. This poses a significant challenge for network’s ability to generalize across different conditions.

**Fig. 7:**
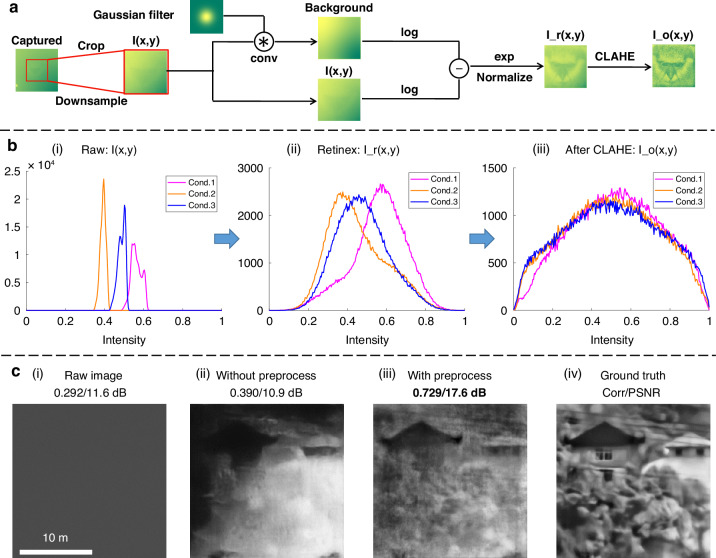
Effectiveness of data processing. **a** Flow chart of the preprocessing method. **b** Histograms of raw images under different conditions at three processing stages. (i), The histograms of the raw images at various concentrations. (ii) The histograms following Retinex-based processing. (iii) The histograms post-CHAHE processing. **c** Ablation study of our processing method, demonstrating the significance of each step in enhancing image restoration quality

To mitigate this challenge, we propose the Retinex-based preprocessing method, design to reduce the domain gap under different experimental conditions. The preprocessing procedure is shown in Fig. 7a. Its objective is to remove the uneven background of the captured and normalize the dynamic range so as to facilitate better feature extraction.

For further analysis, we plot the histograms of images under different condition (three images with varying concentrations) at various stages of preprocessing, shown in Fig. 7b. The initial histogram (i) at the raw stage reveals distinct distribution intervals and shapes, indicating substantial differences in lightness distributions. Direct training on dataset from a specific condition would result in suboptimal reconstruction for images under other conditions, as illustrated by the purple line in Fig. 3. The histogram (ii) post Retinex^49^ processing shows that while differences in lightness distribution intervals have been eliminated, variations in histogram shapes persist. Following additional Contrast Limited Adaptive Histogram Equalization (CLAHE) processing, as shown in (iii), the histograms converge.

The significance of the preprocessing method is underscored by the experimental results shown in Fig. 7c. Without preprocessing, a network trained exclusively on indoor data struggles to accurately reconstruct real-world outdoor objects. In contrast, the application of the proposed preprocessing method significantly enhances the network’s ability to generalize to outdoor conditions, yielding superior reconstruction results. This demonstrates that the preprocessing method effectively address the critical issue of poor generalization due to disparate experimental conditions, enabling the network to focus on fitting reconstruction functions.

### Comparison of different neural networks

In this subsection, we conduct a comparative analysis to evaluate the performance of the proposed Descatter-Net against several other prevalent learning-based methods, including HNN^26^, MulScaleCNN^29^, Unet^44^, AttentionUNet^45^ and SwinIR^46^. These methods are assessed on their ability to perform imaging through a tank of fat emulsion with an optical thickness of 5.5.

The comparison criteria encompass several key metrics: Model Size (quantified by the number of network parameters N_para_), Computational Complexity (measured in terms of floating-point operations: FLOPs), Inference Speed (expressed in frames per second, FPS, based on the same computation platform, the RTX3090), Image Quality (assessed using Corr and PSNR). All neural networks were trained with the same strategy on the same dataset to ensure a fair comparison.

The quantitative results summarized in Table 1 demonstrate the superiority of DescatterNet across various metrics. It achieves the highest image quality with Corr of approximately 0.85 and PSNR of around 18 dB. DescatterNet has a relatively modest model size of less than 2 million parameters and a computational complexity of 10.6 ×10^9^ FLOPs. This efficiency allows for a swift inference speed (up to 338 FPS) that is conducive to real-time incoherent imaging through dynamic scattering media. This capability is further illustrated in the Supplementary Video.

In contrast, models like the transformer-based SwinIR^46^, despite having a smaller model size of 0.14 millions parameters, exhibit higher computational complexity compared to DescatterNet. This results in increased training inference times, with SwinIR achieving only 1.15 FPS in our tests.

In conclusion, we have demonstrated a versatile learning-based method for real-time incoherent imaging through dynamic scattering media: DescatterNet. This method has been effectively demonstrated in both controlled laboratory settings and unpredictable outdoor environments, including conditions where the scattering medium, such as fog, and the real-world objects were not part of the training data. Experimental results suggest that the DescatterNet outperforms other prevalent learning-based and traditional methods across crucial metrics. It excels not only in the quality of reconstructed images but also in its modest computational resource requirements, making it a practical solution for real-time applications.

Despite these advancements, there are still challenges to be addressed in future work. These include: contrast over-enhancement, outdoor large field-of-view image restoration, and the combination of optical filtering methods, and the enhancement of interpretability. We will be focused on tackling these issues, making significant strides towards more reliable and higher-fidelity imaging systems that can be deployed in diverse real-world scenarios.

## Methods and materials

### Experimental setup for acquiring the training dataset

The proposed DescatterNet is trained on a dataset acquired through a homemade scattering environment, a tank with dimensions of 32 cm × 32 cm × 60 cm, filled fat emulsion (Intralipid 20%, Fresenius Kabi). The optical properties of this medium has been well documented^54^. The optical thickness of medium can be adjusted by dropping a certain amount of intralipid into the tank of purified water^29^.

To mimic a non-intrusive imaging mode, the light source is designed to interact with the scattering medium twice. Initially, it illuminates the medium directly, causing light to scatter and subsequentially illuminate the object. The light then propagates back through the scattering medium and is captured by an imaging system, which is comprised of an sCMOS camera (PCO Edge 4.2) and a commercial Nikon lens (AF Nikon 50 mm f/1.8D).

The training set is composed of pair-up images of a number of target objects and the corresponding scattered patterns as seen through the tank of fat emulsion. The ground truth images consists of 1350 images selected from ImageNet^43^ and DIV2K^55^, which are displayed on an e-ink display (10.3-inch, HDMI, e-paper, 1872 × 1404 pixels) during the data acquisition process.

Beyond the training set, the setup is also used to acquire the scattered patterns of “virtual” objects not included in the training set, such as the Pepper, Cameraman images, as well as real-world objects such as the USAF target, china dolls, and a Rubik’s cube.

### Experimental setup for outdoor imaging through fog

One of the key contributions of this work is that our DescatterNet trained on the indoor laboratory environment can be used for outdoor imaging real-world objects through fog. To accommodate the natural fog and to capture real-world objects at a distance, we employ a telescope in place of the optical imaging system used during the indoor dataset acquisition. Our outdoor imaging system was built by integrating an angular selection device (KURIOS-WL1/M, Thorlabs) into a commercial Celestron telescope (CPC1100HD)^52^ which has an angle of view (FOV) of 0.27^o^ [Fig. 5a)]. The angular selection device is crucial for filtering the light based on the angle of incidence. Its transmittance is highly dependent on the angle, dropping sharply as the incident angle of the incoming light increases from 0 to about 6^o^. This means that it only accepts the scattered light impinging the telescope with relatively small angles. We also use a PCO Edge4.2 sCMOS camera to capture the scattered patterns. As shown in Fig. 5b, our outdoor experiment focuses on imaging distant object, such as a house in forest and a villa located 5.9 km away.

### Data preprocessing pipeline

To reduce the domain gap across different scattering conditions, we propose ae data preprocessing method that combines Retinex and CLAHE. This approach minimizes the impact of dataset bias and enhances the network’s ability to generalize to various scattering media and outdoor conditions.

According to the Retinex theory^48,49^, image intensity $I(x,y)$ at each pixel location $(x,y)$ is the product of the reflectance $R(x,y)$ and illumination intensity $L(x,y)$. Retinex-based algorithms aim to remove the illumination function $L(x,y)$ so as to obtain the reflectance $R(x,y)$ of an object in question. Here we adapt this theory for incoherent imaging through scattering media and propose an extended model

$$I\left(x,y\right)=R\left(x,y\right)L\left(x,y\right)T\left(x,y\right)+A(x,y)$$

where $T\left(x,y\right)$ is the transmittance function, which accounts for the attenuation of light as it passes through the scattering medium, and $A(x,y)$ denotes the ambient light or noise that is not related to the object’s reflectance or the illumination source.

Thus, by separating the effects of illumination, reflectance, transmittance, and ambient light, Retinex-based algorithms can attempt to estimate and recover the intrinsic reflectance properties of objects, even in the presence of scattering. The method we employ in this study is

$${I}_{r}\left(x,y\right)=\frac{I(x,y)}{{{\mathscr{F}}}^{-1}\left\{{\mathscr{F}}\left\{I\left(x,y\right)\right\}{\mathscr{\cdot }}{\mathscr{F}}{\mathscr{\{}}g\left(x,y,\sigma \right)\}\right\}+\epsilon \,}$$

where ${\mathscr{F}}$ and ${{\mathscr{F}}}^{-1}$ denote Fourier and inverse Fourier transforms, $\epsilon$ is a renormalization term, $g\left(x,y,\sigma \right)$ represents a Gaussian filter with the standard deviation $\sigma$. is the output image.

The resulting image ${I}_{r}\left(x,y\right)$ is then normalized and further processed using the CLAHE algorithm^50,56^. Unlike traditional histogram equalization, which can over-amplify noise, especially in low contrast regions, CLAHE applies a process of histogram equalization locally, within small, non-overlapping tiles or segments of the image. Each tile is processed independently, which allows for the enhancement of local contrast without introducing artifacts that might be caused by large-scale intensity variations. The equalization respects a predefined contrast limit to prevent noise amplification. After the local equalization, the boundaries between the tiles are smoothly interpolated to ensure a natural transition and to maintain the overall coherence of the image. The result is an image with improved visibility of details and more uniform distribution of intensity levels, which is particularly beneficial for images with non-uniform illumination or in applications where the enhancement of subtle details is crucial.

The complete preprocessing algorithm is shown in the following pseudo-code:

#### Algorithm 1

Preprocessing algorithm

**Input:** *I*_*i*_(*x, y*), raw images with a resolution of 2048 × 2048

1: **Cropping**

2:  *I*_*c*_(*x*, *y*) ← crop the center region of interest

3:  *I* (*x*, *y*) ← resize *I*_*c*_(*x*, *y*) to 448 × 448

4: **Retinex-based**

5: *m*, *n* ← size of *I* (*x*, *y*)

6:  filter: *g*_*m×n*_ (*x*, *y*, σ) ← exp{− [(x − m/2)^2^ + (y − n/2)^2^] / σ^2^}

7:  ***F***_*g*_, ***F***_*I*_ ← $$\boldsymbol{\mathscr{F}}$${*g*}, $$\boldsymbol{\mathscr{F}}$${*I*}

8:  background: *bg*(*x*,*y*) ← $$\boldsymbol{\mathscr{F}}$$^–1^{ ***F***_*g*_ · ***F***_*I*_ }

9:  retinex: *I*_*r*_(*x*, *y*) ← *I* (*x*, *y*) / *bg*(*x*,*y*) ← exp[ln(*I*(*x*,*y*)–ln(*bg*(*x,y*))]

10: normalize: *I*_*r*_(*x*, *y*) ← [*I*_*r*_ − min(*I*_*r*_)]/[max(*I*_*r*_) − min(*I*_*r*_)]

11: **CLAHE**

12: *I*_*tile*_(*x*,*y*) ← divide *I*_*r*_(*x*, *y*) into 8 × 8 tiles

13: *h*_*tile*_ ← contrast limit histogram of each tile

14: ${I}_{{tile}}^{{\prime} }(x,y)$ ← transform pixel value of each *I*_*tile*_(*x*,*y*)

15: *I*_*o*_(*x*,*y*) ← interpolate($${I}_{{tile}}^{{\prime} }(x,y)$$)

**Output:** *I*_*o*_(*x*,*y*), preprocessing image

### DescatterNet architecture

The DescatterNet is composed of two primary components: an Encoder and a Decoder. The Encoder accepts an input of size 448 × 448 × 1 pixels and sequentially performs four down-sampling operations through max-pooling, resulting in five feature layers at varying scales. Following each down-sampling operation, the number of channels in the convolutional layers is incremented by a factor of two. At each scale, the initial feature layer is processed through a convolutional block that comprises two 3 × 3 convolutional layers with *N* channels, succeeded by a batch normalization layer and a rectified linear unit (ReLU) activation function, as depicted by the blue legend in Supplementary Fig. S1 in the Supplementary material. To bolster the network’s generalization, a dropout layer with a rate of 0.1 is integrated at the end of each scale’s feature layer.

The Decoder reconstructs the image through four up-sampling operations, realized by transposed convolutions, also known as deconvolutions. Post each up-sampling step, the Decoder concatenates the final feature layer from the corresponding scale in the Encoder, as indicated by the skip connections in the legend. These skip connections facilitate the incorporation of fine-grained information from the Encoder into the reconstruction process. The concatenated feature layer then proceeds through another convolutional block and a dropout layer. The network culminates with a convolutional layer designed for grayscale images, featuring one channel with an output size of 448 × 448 × 1. For color images, the channel count in the final layer is adjusted to 3. The output of the network is regulated to a range between 0 and 1 by employing a sigmoid activation function.

To determine the number of channels, we conducted comparative experiments with various basic channel numbers, including 1, 2, 4, 8, 16, 32, and 64. The results of these comparisons are list in Supplementary Table S1 in the Supplementary material. We observed that the inference speed of the UNet does not increase when the basic channel number falls below 16, as the computational cost is then predominantly determined by factors other than network depth. Furthermore, experiments demonstrated that a higher number of network layers does not necessarily yield superior performance; for instance, networks with 64 and 32 channels underperformed compared to those with 16.

After evaluating multiple neural networks based on memory usage, inference speed, and recovery performance, we selected the optimal UNet with a basic channel number of 16 for our DescatterNet. This choice was made because it delivers strong performance across all evaluated criteria. This selection offers significant reference value for extensive research, as this network can be directly applied to validate our learning-based methods.

### DescatterNet training

The mean square error (MSE) is used as the loss function to train the DescatterNet:

$$L=\mathop{{\rm{argmin}}}\limits_{\theta }\frac{1}{N}\mathop{\sum }\limits_{i=1}^{N}{{||}{G}_{\theta }\left({I}_{i}\right)-{R}_{i}{||}}^{2}$$

where ${G}_{\theta }$ represents the network with parameter $\theta$, ${I}_{i}$ is the input image. ${R}_{i}$ is the corresponding label (ground truth), and $N$ is the size of the dataset. The MSE loss function can train an effective network more efficiently than other loss functions.

The first 1300 pairs of data from the e-ink display were allocated to form the training set, with 1200 used for training and 100 for validation purposes. The remaining data comprised the test set. The network was initialized with a learning rate of 0.01. If the validation loss plateaus for 50 epochs without improvement, the learning rate is reduced to one-tenth of its original value, with a minimum threshold set at 0.00001. Training was conducted for 200 epochs using a batch size of 8 (Please refer to Supplementary Fig. S2 in the Supplementary material for the loss curve). The Adam optimizer was employed for the optimization process, and the model exhibiting the optimal performance on the validation set was preserved.

### Supplementary information

Supplementary Information for Learning-based real-time imaging through dynamic scattering media

Video recording for incoherent imaging through a tank of fat emulsion

## Data Availability

Data will be available at https://github.com/SituLab/DescatterNet-for-unseen-real-world-objects when the paper is officially published.

## Code availability

Code will be available at https://github.com/SituLab/DescatterNet-for-unseen-real-world-objects when the paper is officially published.
