# Supplementary material for: Learning-based real-time imaging through dynamic scattering media
Source: Light Sci Appl. 2024 Aug 16;13:194. doi: 10.1038/s41377-024-01569-0 (PMC11329739; doi:10.1038/s41377-024-01569-0)
Supplement: Supplementary file 1 — Supplementary Information for Learning-based real-time imaging through dynamic scattering media [file 41377_2024_1569_MOESM1_ESM.docx]

**Supplementary Information**

**Learning-based real-time imaging through dynamic scattering media**

**Haishan Liu**^1,2^, **Fei Wang**^1^, **Ying Jing**^1^, **Xianzheng Ma**^3^, **Siteng Li**^1^, **Yaoming Bian**^1^, and **Guohai Situ**^1,2,4,*^

^1^Shanghai Institute of Optics and Fine Mechanics, Chinese Academy of Sciences, Shanghai, 201800, China

^2^Center of Materials Science and Optoelectronics Engineering, University of Chinese Academy of Sciences, Beijing, 100049, China

^3^Department of Engineering Science, University of Oxford, Oxford, UK

^4^Hangzhou Institute for Advanced Study, University of Chinese Academy of Sciences, Hangzhou 310024, China

^*^corresponding.author: [ghsitu@siom.ac.cn](mailto:ghsitu@siom.ac.cn),

**1. Data acquisition**

We have collected extensive data, including images from an e-ink display and real-world objects. The e-ink display, which possesses optical properties akin to those of real-world objects and can cycle through images, allows us to flexibly address the core challenge of data collection in actual scattering environments.

The data were acquired using an automated control program that simultaneously manages the e-ink display and the imaging system. Initially, the e-ink display is prompted to present an image from the dataset, followed by the camera's capture of that image. This sequence is repeated for each image at varying concentrations of the scattering medium, ensuring a one-to-one collection of corresponding scattered and clear images.

We chose public datasets, ImageNet and DIV2K, to display diverse real scenes on the e-ink display. These datasets are rich in natural scenes, which helps to circumvent the issue of oversimplified features found in datasets such as MNIST. Due to constraints in acquisition time and computational memory, we selected 675 images from each dataset. For testing purposes, we selected common 2D images and 3D real-world objects, including Pepper, Cameraman, USAF, Lena, toy models, and a Rubik's Cube, among others. In total, we have amassed 1368 sets of data and a video for each experimental condition:

**Condition 1: Varying Optical Thickness**. We introduced fat emulsion solution to pure water within a tank of dimensions 17 cm × 32 cm × 60 cm to create experimental conditions with different levels of scattering. These conditions were calibrated by optical thickness, with final concentrations set at 0 ml, 0.6 ml, 1.2 ml, 1.8 ml, 2.8 ml, 3.2 ml, and 3.6 ml, corresponding to optical thicknesses of 0.53, 1.67, 2.97, 4.20, 5.51, 6.35, 7.19, and 7.92, respectively. At the highest concentrations, object details in the captured images were challenging to discern even after enhancement, enabling us to explore the upper limits of our method's performance.

**Condition 2: Diverse Scattering Media.** Beyond the fat emulsion solution experiments, we also gathered data from other scattering media—milk solution and artificial fog. We created a milk-scattering solution by diluting milk and generated dense fog within a glass tank using an ultrasonic fog machine (model SG-06D/10D).

**Condition 3: Different Displays.** As a control experiment, we collected data displayed on a spatial light modulator (SLM) and compared the performance of networks trained on SLM and e-ink display datasets.

**Condition 4: Outdoor Fog Data**. To assess the practical performance of our descattering method, we collected outdoor fog data using our outdoor experimental setup.

**2. Measurement of Optical Thickness**

Here we use Optical thickness (OT) as a metric to evaluate the degree of environment scattering, which is characterized an exponential decrease in signal energy. According to the definition of OT, once we measure the optical power $P_{in}$ when the light enters the scattering medium and the optical power$P_{out}$ when the light exists, the OT can be calculated as

$$\mathrm{OT}=\ln\frac{P_{out}}{P_{in}}$$

To characterize the degree of scattering of the media we attempt to image through, we employ a 532nm laser beam to illuminate the scattering medium, and subsequently, we use an optical power meter to measure $P_{in}$ and $P_{out}$. The results from multiple repeated measurements are consistent, indicating that the change in optical thickness is linearly related to the change in concentration.

**3. The DescatterNet**

**3.1 Architecture**


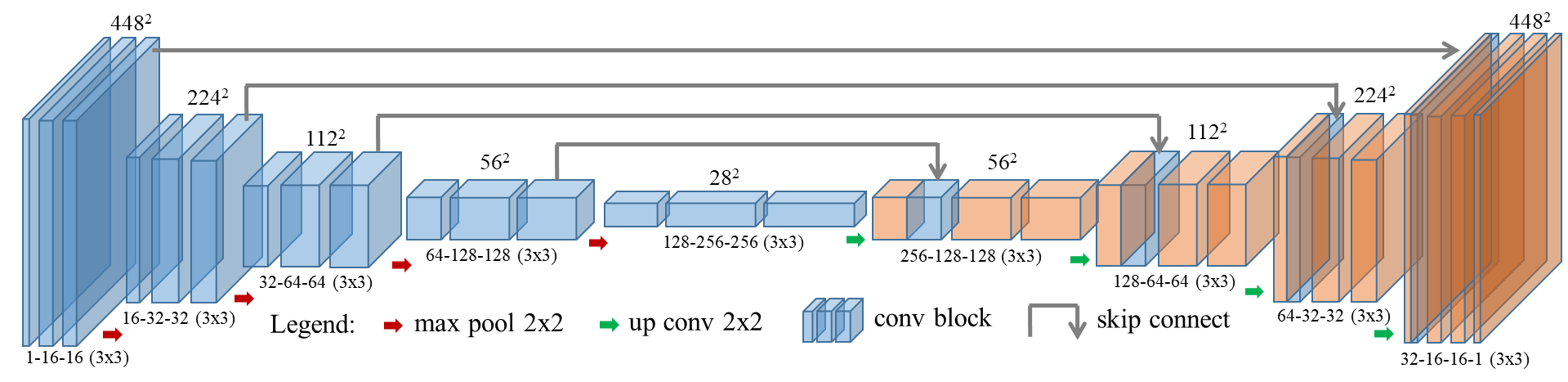


**Fig.S1 DescatterNet architecture**.

**3.2 The determination of channel number**

**Table S1** Performance Comparison in terms of basic channel number

| Index | Method | N_para_ | FLOPs | Speed(RTX3090) | m_ssim | m_psnr |
| --- | --- | --- | --- | --- | --- | --- |
| 1 | UNet1 | 0.01M | 0.05G | 358.61 fps | 0.3091 | 17.87 |
| 2 | UNet2 | 0.03M | 0.18G | 361.58 fps | 0.3120 | 17.93 |
| 3 | UNet4 | 0.12M | 0.69G | 352.65 fps | 0.3166 | 17.98 |
| 4 | UNet8 | 0.49M | 2.69G | 346.84 fps | 0.3181 | 18.00 |
| 5 | **UNet16** | 1.94M | 10.59G | 338.62 fps | **0.3184** | **18.00** |
| 6 | UNet32 | 7.77M | 42.03G | 157.57 fps | 0.3147 | 17.93 |
| 7 | UNet64 | 31.04M | 167.51G | 65.02 fps | 0.3048 | 17.80 |

**3.3 Convergence behavior**

**Fig. S2** Loss curve during the training and validation processes.
